# Single Herbal Medicine for Insulin Resistance: Protocol for a Systematic Review and Meta-Analysis of Randomized Clinical Trials

**DOI:** 10.2196/68915

**Published:** 2025-06-10

**Authors:** Jialing Zhang, Zhinan Zhang, Hoi Ki Wong, Shuyan Zhong, Zhilin Lin, Zhaoxiang Bian

**Affiliations:** 1 Vincent V.C. Woo Chinese Medicine Clinical Research Institute School of Chinese Medicine Hong Kong Baptist University Hong Kong China; 2 School of Chinese Medicine Hong Kong Baptist University Hong Kong China; 3 School of Traditional Chinese Medicine Southern Medical University Guangzhou China; 4 Chinese EQUATOR Centre Hong Kong Baptist University Hong Kong China; 5 Centre for Chinese Herbal Medicine Drug Development Hong Kong Baptist University Hong Kong China

**Keywords:** insulin resistance, single herbal medicine, systematic review, homeostatic model assessment for insulin resistance, oral glucose tolerance test

## Abstract

**Background:**

Insulin resistance (IR) is a central factor in the pathogenesis and progression of metabolic disorders, such as type 2 diabetes mellitus and obesity. Chinese herbal medicine (CHM) has been investigated as a potential therapy to enhance insulin sensitivity. Compared to multiherb formula therapy, single-herb therapy provides a clearer understanding of its pharmacological effects and mechanisms of action. A systematic review of the available evidence is needed to elucidate the potential effectiveness and harm of single CHMs for IR.

**Objective:**

This study aims to conduct a systematic review and meta-analysis to evaluate the potential effectiveness and harm of single herbs for the treatment of IR, thereby providing a clearer understanding of the efficacy and safety profiles of single CHMs.

**Methods:**

A systematic review and meta-analysis, in accordance with the PRISMA (Preferred Reporting Items for Systematic Reviews and Meta-Analyses) 2020, will be conducted to evaluate the efficacy and safety of single herbs for IR. Various databases, such as Cochrane Central Register of Controlled Trials, MEDLINE, Embase, Allied and Complementary Medicine Database, China National Knowledge Infrastructure, Chinese BioMedical Literature Database, and the Wanfang Database, will be searched. Randomized controlled trials comparing single herbs or extracts originated from single herbs with a placebo or no treatment for adults diagnosed with IR-related diseases (eg, type 2 diabetes mellitus and obesity) will be included. A total of 2 researchers will independently perform study selection, data extraction, and quality assessment. The risk of bias (RoB) tool will be used to assess the quality of included studies. The overall certainty of evidence will be assessed using Grading of Recommendations Assessment, Development, and Evaluation (GRADE). The primary outcome will include measurements that assess IR, such as the hyperinsulinemic-euglycemic clamp, homeostatic model assessment, insulin sensitivity index, and oral glucose tolerance test. Secondary outcomes will include adverse events. Meta-analysis will be performed with RevMan (version 5.4; The Cochrane Collaboration). The heterogeneity of the synthetic data will be assessed using the chi-square test and the *I*^2^ statistic.

**Results:**

Based on the data on IR-associated outcomes (eg, hyperinsulinemic-euglycemic clamp, homeostatic model assessment, insulin sensitivity index, and oral glucose tolerance test) and adverse event rates, this study will provide an evidence-based review and high-quality synthesis regarding the efficacy and safety of single CHMs for IR.

**Conclusions:**

This systematic review and meta-analysis will rigorously synthesize existing evidence to clarify the efficacy and safety of single CHMs in ameliorating IR, offering critical insights for their integration into evidence-based therapies for metabolic disorders. By focusing on single-herb therapy, the findings may promote the application of CHM for IR, bridge the gap between traditional applications and evidence-based practice, and ultimately optimize the role of CHM in integrative metabolic health management.

**Trial Registration:**

PROSPERO CRD42024589362; https://www.crd.york.ac.uk/PROSPERO/view/CRD42024589362

**International Registered Report Identifier (IRRID):**

PRR1-10.2196/68915

## Introduction

Insulin resistance (IR), defined as an impaired biological response to insulin stimulation of target tissues, primarily involves liver, muscle, and adipose tissue [1-3]. IR is the consequence of the inhibition of the insulin signaling pathway that may result from mutations or posttranslational modification of the insulin receptor or the downstream effector molecules [4]. Consequently, insulin action is disrupted in insulin-sensitive tissues, leading to abnormal glucose metabolism and dysfunction in insulin secretion [5]. IR is present in one-quarter of the general population, predisposing these people to a wide range of diseases [6]. IR is a central component of type 2 diabetes mellitus (T2DM) and is associated with various pathological states, such as metabolic syndrome, obesity, polycystic ovary syndrome, nonalcohol fatty liver diseases, hypertension, and cardiovascular diseases (CVDs) [7-9]. The rising incidence of IR and its associated complications pose a significant medical and socioeconomic burden [10]. Improving insulin sensitivity may provide a therapeutic strategy for controlling T2DM and IR-associated diseases.

Lifestyle modifications (eg, physical exercise and dietary adjustments), medications, and complementary and alternative medicine are commonly used for managing IR [11,12]. Chinese herbal medicine (CHM), a crucial element of complementary and alternative medicine, has been investigated as a potential therapy to enhance insulin sensitivity [13]. CHM encompasses a wide range of applications, including single herbs, herbal active components, and herbal formulas [14]. While herbal formulas combine multiple herbs, single herbal medicine uses a single herb (or its extract), offering a clearer understanding of its pharmacological effects and mechanisms of action. Randomized clinical trials have demonstrated that CHM prescriptions exhibit comparable efficacy to metformin in reducing homeostatic model assessment for insulin resistance (HOMA-IR) among patients with T2DM [15,16]. A systematic review of 14 studies (n=1255) found that berberine significantly reduced HOMA-IR in individuals with T2DM compared to control, with a reduction of 0.71 (95% CI –1.03 to –0.39) [17]. Given the increasingly widespread use of CHM for treating IR-associated diseases, concerns about its effectiveness and safety are increasing. A systematic review of the available evidence is needed to elucidate the potential effectiveness and harm of single CHMs for IR. Due to the large number of mixed Chinese herbal prescriptions presenting a great challenge to the evaluations, this systematic review and meta-analysis focuses on single CHMs and aims to evaluate the efficacy and safety of managing IR.

## Methods

### Study Registration

The protocol of this meta-analysis has been registered in PROSPERO (International Prospective Register of Systematic Reviews) with the registration number CRD42024589362. This review will be conducted and reported in line with the PRISMA (Preferred Reporting Items for Systematic Reviews and Meta-Analyses) 2020 statement [18].

### Eligibility Criteria

#### Study Types

The included studies must be randomized controlled trials regardless of blinding method and publication status. Nonrandomized trials, quasi-experimental studies, animal experiments, editorials, letters, commentaries, study protocols, reviews, case reports, and conference abstracts will be excluded.

#### Participants

The included studies enrolled adults (18 years old) diagnosed with IR-related diseases, such as T2DM, obesity, polycystic ovary syndrome, hyperlipidemia, metabolic syndrome, and CVDs [19].

#### Interventions and Controls

Interventions will include single herbs or extracts originating from any single herb regardless of their compositions or administration route. Eligible single herb is based on traditional Chinese medicine theory and its form of use must have been listed in the latest version of the Chinese Pharmacopoeia (2020) or the National Essential Drug List (2018) of the People’s Republic of China. The comparators include placebo or no treatment and allow cointerventions administrated in both intervention and control groups.

#### Outcomes

Studies will be included if they reported one of the following predefined outcomes: hyperinsulinemic-euglycemic clamp (HEC), insulin suppression test (IST), insulin tolerance test (ITT), intravenous glucose tolerance test (IVGTT), oral glucose tolerance test (OGTT), oral glucose insulin sensitivity index (OGSI), continuous infusion of glucose with model assessment (CIGMA), minimal modeling, fasting insulin (FINS), HOMA-IR, quantitative insulin-sensitivity check index (QUICKI), insulin sensitivity index, homeostasis model assessment-β (HOMA-β), 2-hour insulin, acute insulin response (AIR), disposition index, C-peptide level, insulin-to-glucose ratio, triglyceride glucose index (TyG index), triglyceride/high-density lipoprotein cholesterol (TG/HDL-C) ratio, TyG-BMI, metabolic score for insulin resistance (METS-IR), McAuley index, and glycated hemoglobin [20-23]. Secondary outcomes will include adverse events.

### Database and Search Strategies

The following electronic databases will be searched: Cochrane Central Register of Controlled Trials (CENTRAL, Ovid), MEDLINE (Ovid, from 1946 to present), Embase (Ovid, from 1974 to present), Allied and Complementary Medicine Database (Ovid, from 1985 to present), China National Knowledge Infrastructure (from 1949 to present), Chinese BioMedical Literature Database (from 1949 to present), and Wanfang Database (from 1985 to present). Search terms include (traditional medicine OR oriental medicine OR medicinal plants OR phytotherapy OR Chinese herbal drugs OR plant extracts OR herbal medicine) and (insulin resistance OR insulin sensitivity OR glucose intolerance OR glucose tolerance OR impaired glucose OR impaired glycaemia OR metabolic syndrome OR prediabetic state). The search strategy will be developed and modified for different databases (Table S1 in Multimedia Appendix 1). We will apply the Cochrane sensitivity-maximizing randomized controlled trial filter with adaptations [24]. There will be no restriction on the language. Gray literature will be searched with Google. We will also use the ClinicalTrials.gov and World Health Organization International Clinical Trials Registry Platform (ICTRP) Search Portal to identify ongoing or unpublished trials. In order to gather missing information and details of ongoing trials, we will reach out to the authors for clarification. We will further review the reference lists of included studies and any relevant systematic reviews to identify additional references. The screening process of the searched studies will be conducted according to the PRISMA flow diagram (for checklist, see Multimedia Appendix 2) [25].

### Study Selection

Literature retrieved citations will be managed by EndNote (version 20; Clarivate) software. Eligibility evaluation will be done by title and abstract reviews and when abstracts do not provide enough information, the full text of the paper will be retrieved for evaluation. This will be performed independently by 2 reviewers. Discrepancies will be resolved by consensus and arbitration by a third reviewer when necessary.

### Data Extraction and Management

A data extraction form will be constructed and include general information (author, year, title, journal, language, and country), participant characteristics, and study design (sample size, age, gender, IR-related diseases, diagnosis criteria, group assignment, and blinding), treatment regimen (dosage, form, administration route, duration, and frequency), clinical outcomes, adverse events, and assessments of risk of bias. Before implementing it for all included studies, we will conduct a pilot test using at least 1 study from the review. A total of 2 review authors will independently extract study characteristics from the included studies.

### Quality Assessment

The risk of bias (RoB) of the included studies will be adopted by 2 reviewers independently using the Cochrane revised tool for randomized trials (RoB 2) [24]. Each trial will be scored as either high, low risk, or some concerns for 5 items: randomization process, deviations from intended interventions, missing outcome data, measurement of the outcome, and selection of the reported result. When there are disagreements, a third reviewer will be consulted.

The overall certainty of evidence will be assessed by 2 assessors using Grading of Recommendations Assessment, Development, and Evaluation (GRADE), as outlined by the GRADE Working Group [26]. The 5 items will be investigated, including limitations in study design, inconsistency, imprecision, indirectness, and publication bias. The certainty of evidence will be summarized in 4 categories: high, moderate, low, and very low certainty.

### Data Synthesis and Analysis

We will perform statistical analysis using the RevMan (version 5.4; The Cochrane Collaboration) software. We will calculate the mean difference or risk ratio with 95% CI, separately, for continuous and dichotomous outcomes. Data heterogeneity will be assessed using the chi-square test and the *I*^2^ statistic. A fixed-effects model will be used to analyze pooled effects when heterogeneity is not significant (*P*≥.10 or *I*^2^≤50%); a random-effects model will be used when heterogeneity is statistically significant (*I*^2^>50% or *P*<.10). We plan to conduct the following subgroup analyses: single herbal medicine versus placebo, single herbal medicine versus no treatment, and single herbal medicine combined with cointervention versus cointervention alone. Sensitivity analysis will be conducted by excluding trials with a high RoB in more than 2 domains or with a dropout rate exceeding 20%. Egger regression test and funnel plot (if more than 10 trials are included) will be performed to assess the publication bias of the included studies.

Missing or ambiguous data will be addressed by contacting the first or corresponding authors via email twice (at 2-wk intervals) to request additional information. In cases where data remain unavailable despite repeated correspondence attempts, the affected studies will be excluded from quantitative synthesis. For included studies with partial missing data, intention-to-treat analysis data will be used.

## Results

The study will be conducted following the methodology outlined in the Cochrane Handbook for Systematic Reviews of Interventions. A total of 2 researchers will independently perform the study selection, data extraction, and quality assessment. By focusing on IR-associated outcomes (eg, HEC, IST, ITT, and OGTT) and adverse events, this study aims to provide an evidence-based review and high-quality synthesis regarding the efficacy and safety of single herbs for IR.

## Discussion

### Overview

IR is a central factor in the pathogenesis and progression of metabolic disorders, including T2DM, obesity, and hypertension [27]. Furthermore, a strong association has been established between IR and an increased risk of developing cardiovascular diseases [28]. The intricate mechanisms underlying IR may involve complex interplay among inflammation, endoplasmic reticulum stress, oxidative stress, and mitochondrial dysfunction [29-32]. CHM has emerged as a potential therapeutic modality for enhancing insulin sensitivity [13,33]. Numerous CHMs have demonstrated therapeutic effects in improving insulin sensitivity in peripheral tissues, enhancing glucose-stimulated insulin secretion, promoting browning of white adipose tissue, and lowering glucose and lipid levels [34-37]. The growing use of CHM for improving insulin sensitivity underscores the urgent need for a thorough assessment of its effectiveness and safety.

### Anticipated Principal Findings

This systematic review and meta-analysis will synthesize existing evidence to comprehensively evaluate the therapeutic efficacy and safety profiles of single CHMs for insulin IR. Subgroup analyses will compare single herbs against placebo or no treatment and assess efficacy variations across IR-associated conditions (eg, T2DM and obesity), providing important insights into context-specific therapeutic potential. In addition, critical appraisal of the study and reporting quality in included trials will identify methodological limitations in current research, informing recommendations for future trial design and transparency.

### Outcome Selection

While the HEC remains the gold standard for quantifying IR due to its direct measurement of insulin-mediated glucose uptake [38-40], its clinical utility is limited by technical complexity and invasiveness [41]. Consequently, multiple validated markers, such as fasting-based (eg, HOMA-IR and QUICKI), dynamic tests (eg, OGTT and IVGTT), and composite indices (eg, TyG index and METS-IR), are widely adopted in clinical research [20-23], Among these, HOMA-IR is prioritized in observational and interventional studies due to its simplicity and reproducibility [38]. To ensure methodological inclusivity, this review will incorporate all IR-associated outcomes.

### Strengths and Limitations

This represents the first systematic evaluation of all single herbs and herbal extracts for IR, addressing a critical gap left by prior reviews focused on multiherb formulas [42,43] or isolated compounds [44,45]. While single-herb analyses deviate from traditional multiherb clinical practice, they provide clarity in therapeutic efficacy essential for evidence-based integration into IR management protocols. However, heterogeneity in treatment details (eg, administration routes and treatment duration) and the potential inclusion of low-quality studies may limit the generalizability of findings.

### Future Directions

This study may inform the potential of single CHMs as alternative therapies for IR, bridging traditional knowledge with clinical needs. Identified high-priority herbs should undergo rigorous preclinical validation (eg, pharmacokinetic studies, in vivo mechanistic models) followed by confirmatory clinical trials with rigorous design. Concurrently, the quality assessment framework established here may guide future trial designs to minimize bias and enhance reproducibility.

### Conclusion

This systematic review and meta-analysis will rigorously synthesize existing evidence to clarify the efficacy and safety of single CHMs in ameliorating IR, offering critical insights for their integration into evidence-based therapies for metabolic disorders. By focusing on single-herb therapy, the findings may promote the application of CHM for IR, bridge the gap between traditional applications and evidence-based practice, and ultimately optimize the role of CHM in integrative metabolic health management.

### Dissemination

Results will be disseminated through peer-reviewed journals and presentations at international conferences focused on metabolic disorders, ensuring accessibility to clinicians, researchers, and policy makers.

## Appendix

Multimedia Appendix 1 Literature search strategy.

Multimedia Appendix 2 PRISMA-P (Preferred Reporting Items for Systematic Reviews and Meta-Analyses Protocols) 2015 checklist.

## Abbreviations

AIR: acute insulin response
CHM: Chinese herbal medicine
CIGMA: continuous infusion of glucose with model assessment
CVD: cardiovascular disease
FINS: fasting insulin
GRADE: Grading of Recommendations Assessment, Development, and Evaluation
HEC: hyperinsulinemic-euglycemic clamp
HOMA-β: homeostasis model assessment-β
HOMA-IR: homeostatic model assessment for insulin resistance
ICTRP: International Clinical Trials Registry Platform
IR: insulin resistance
IST: insulin sensitivity index
ITT: insulin tolerance test
IVGTT: intravenous glucose tolerance test
METS-IR: metabolic score for insulin resistance
OGSI: oral glucose insulin sensitivity index
OGTT: oral glucose tolerance test
PRISMA: Preferred Reporting Items for Systematic Reviews and Meta-Analyses
PROSPERO: International Prospective Register of Systematic Reviews
QUICKI: quantitative insulin-sensitivity check index
RoB: risk of bias
T2DM: type 2 diabetes mellitus
TG/HDL-C: triglyceride/high-density lipoprotein cholesterol
TyG index: triglyceride glucose index

## Data Availability

The datasets generated or analyzed during this study are available from the corresponding author on reasonable request.
