# Supplementary material for: Single Herbal Medicine for Insulin Resistance: Protocol for a Systematic Review and Meta-Analysis of Randomized Clinical Trials
Source: JMIR Res Protoc. 2025 Jun 10;14:e68915. doi: 10.2196/68915 (PMC12188135; doi:10.2196/68915)
Supplement: Multimedia Appendix 1 [file resprot_v14i1e68915_app1.docx]

# **Table S1. Literature search strategy**

| **CENTRAL, MEDLINE(R), Embase via Ovid** | |
| --- | --- |
| 1 | exp Medicine, Herbal/ |
| 2 | exp Plants, Medicinal/ |
| 3 | exp Medicine, Traditional/ |
| 4 | exp Drugs, Chinese Herbal/ |
| 5 | exp oriental medicine/ |
| 6 | exp phytotherapy/ |
| 7 | exp plant extracts/ |
| 8 | (chinese adj3 medic$).tw. |
| 9 | (traditional adj3 medic$).tw. |
| 10 | (orient$ adj3 medic$).tw. |
| 11 | (herb$ adj3 medic$).tw. |
| 12 | (chinese adj3 formula$).tw. |
| 13 | or/1-12 |
| 14 | exp Glucose Tolerance Test/ |
| 15 | exp Glucose Intolerance/ |
| 16 | exp Diabetes Mellitus, Type 2/pc [Prevention & Control] |
| 17 | exp Insulin Resistance/ |
| 18 | exp insulin sensitivity/ |
| 19 | exp Metabolic Syndrome/ |
| 20 | exp Prediabetic State/ |
| 21 | (glucose adj3 (intolerance or tolerance test*)).tw,ot. |
| 22 | (impaired fasting adj3 (glucose or glyc?emia*)).tw,ot. |
| 23 | (impaired glucose adj3 (toleran* or stat* or respons* or control* or regul* or metab* or homeost*)).tw,ot. |
| 24 | (reduced glucose adj3 (metab* or toleran*)).tw,ot. |
| 25 | (pr?ediabet* or pr?e diabet*).tw,ot. |
| 26 | (metabolic syndrom* or syndrome).tw,ot. |
| 27 | ((borderline or mild) adj3 diabet*).tw,ot. |
| 28 | insulin resistan*.tw,ot. |
| 29 | ((impaired or reduced) adj3 insulin secret*).tw,ot. |
| 30 | or/14-29 |
| 31 | exp Randomized Controlled Trial/ |
| 32 | Controlled Clinical Trial.pt. |
| 33 | (randomized or randomized).ab,ti. |
| 34 | placebo.ab,ti. |
| 35 | drug therapy.fs. |
| 36 | randomly.ab,ti. |
| 37 | trial.ab,ti. |
| 38 | groups.ab,ti. |
| 39 | or/31-38 |
| 40 | 13 and 30 and 39 |
| **AMED via Ovid** | |
| 1 | exp herbs/ |
| 2 | exp plant extracts/ |
| 3 | exp plants medicinal/ |
| 4 | exp traditional medicine chinese/ |
| 5 | exp drugs chinese herbal/ |
| 6 | exp phytotherapy/ |
| 7 | (chinese adj3 medic$).tw. |
| 8 | (traditional adj3 medic$).tw. |
| 9 | (orient$ adj3 medic$).tw. |
| 10 | (herb$ adj3 medic$).tw. |
| 11 | (chinese adj3 formula$).tw. |
| 12 | or/1-11 |
| 13 | exp Insulin Resistance/ |
| 14 | (glucose adj3 (intolerance or tolerance test*)).tw. |
| 15 | (impaired fasting adj3 (glucose or glyc?emia*)).tw. |
| 16 | (impaired glucose adj3 (toleran* or stat* or respons* or control* or regul* or metab* or homeost*)).tw. |
| 17 | (reduced glucose adj3 (metab* or toleran*)).tw. |
| 18 | (pr?ediabet* or pr?e diabet*).tw. |
| 19 | (metabolic syndrom* or syndrome).tw. |
| 20 | ((borderline or mild) adj3 diabet*).tw. |
| 21 | insulin resistan*.tw. |
| 22 | ((impaired or reduced) adj3 insulin secret*).tw. |
| 23 | or/13-22 |
| 24 | exp Randomized Controlled Trials/ |
| 25 | Controlled Clinical Trial.pt. |
| 26 | (randomized or randomized).ab,ti. |
| 27 | placebo.ab,ti. |
| 28 | randomly.ab,ti. |
| 29 | trial.ab,ti. |
| 30 | groups.ab,ti. |
| 31 | or/24-30 |
| 32 | 12 and 23 and 31 |
| **CNKI** | |
| 1 | SU=('胰岛素抵抗'+'葡萄糖耐量试验'+'糖耐量试验'+'葡萄糖耐受不良'+'糖尿病前期'+'代谢综合征'+'高血糖') AND SU=('中医'+'中医药'+'中华医药'+'草药'+'中草药'+'中药'+'中药材'+'汤剂'+'方剂'+'中成药'+'注射剂 ') AND SU=('临床试验'+'随机'+'对照'+'随机试验'+'随机对照试验'+'临床研究'+'临床观察 '+'疗效评价') |
| **CBM** | |
| 1 | (胰岛素抵抗 or 葡萄糖耐量试验 or 糖耐量试验 or 葡萄糖耐受不良 or 糖尿病前期 or 代谢综合征 or 高血糖) AND (中医药 or 草药 or 中药 or 汤剂 or 方剂 or 中成药 or 注射剂) AND (临床试验 or 随机 or 对照 or 随机试验 or 随机对照试验 or 临床研究 or 临床观察 or 疗效评价) |
| **Wanfang** | |
| 1 | (主题="胰岛素抵抗" OR 主题="葡萄糖耐量试验" OR 主题="糖耐量试验" OR 主题="葡萄糖耐受不良" OR 主题="糖尿病前期" OR 主题="代谢综合征" OR 主题="高血糖") AND (主题="中医药" OR 主题="草药" OR 主题="中药" OR 主题="汤剂" OR 主题="方剂" OR 主题="中成药" OR 主题="注射剂") AND (主题="临床试验" OR 主题="随机" OR 主题="对照" OR 主题="随机试验" OR 主题="随机对照试验" OR 主题="临床研究" OR 主题="临床观察" OR 主题="疗效评价") |
